# Complex decision making in a patient with lung cancer with incidentally found fast-growing atrial mass

**DOI:** 10.1186/s40959-024-00219-z

**Published:** 2024-05-18

**Authors:** Prince Otchere, Stella Pak, Juan Ulloa-Rodriguez, Maria Fierro, Aditi Sharma, Tevonne Poku, Brandon Kofi-Obeng, Eric Yang, Keerthi Thallapureddy

**Affiliations:** 1 Department of Cardio-Oncology, University of Texas Health Science Center, San Antonio, TX USA; 2 Department of Neurology, Albany Medical Center, 43 New Scotland Ave, Albany, NY 12208 USA; 3 Department of Cardiology, University of Texas Health Science Center, San Antonio, TX USA; 4 Department of Medicine, University of Texas Health Science Center, San Antonio, TX USA; 5 Department of Medicine, Texas A&M University, College Station, TX USA; 6 Department of Pathology, University of Texas Health Science Center, San Antonio, TX USA

**Keywords:** Adenocarcinoma of the lungs, Cardiac metastasis, Left atrial mass, Cardiac myxoma, Cardiac thrombi

## Abstract

Atrial myxomas are typically found in the left atrium and are the most common among overall rare cardiac tumors. Herein, we describe the clinical course of a 72-year-old female with non-small cell lung adenocarcinoma found to have an atrial mass during an imaging for evaluation for lung cancer progression. Differentiating between distinct types of masses can pose a challenge to the treatment team especially in the setting of exiting malignancy. This case demonstrates the complex decision making involved in the diagnosis, and timing of intervention to remove atrial mass in patients with frailty and a fast-growing cardiac mass.

**Supplementary Information:**

The online version contains supplementary material available at 10.1186/s40959-024-00219-z.

## Introduction

Differentiating a secondary cardiac tumor from primary cardiac tumor or thrombus is a diagnostic conundrum and a challenging clinical task. State-of-the-art imagining technologies with echocardiogram and magnetic resonance imaging can shed a light on this arduous clinical journey. The incidence of cardiac metastasis is reported to range from 2.3 to 18.3% in various types of cancer [1]. The types of cancer known to be associated with cardiac metastasis are pleural mesothelioma, melanoma, and lung cancers [2, 3]. Accurately establishing the sites of metastasis is crucial in developing an effective therapeutic regimen in cancer.

Cardiac myxoma is a common primary cardiac neoplasm in adults. The gelatinous and fragile composition of cardiac myxomas and their location make them prone to break off to embolize into various parts of the body [4]. Other possible complications of cardiac myxoma include mitral valve obstruction, congestive heart failure, and coronary artery disease [5]. Thus, it is critical to discern the nature of the mass and distinguish a cardiac myxoma from other pathologies, such as metastasis, thrombosis, lymphoma, lipoma, or fibromyoma [6, 7]. Herein, we present a case of a patient with recurrent and metastatic adenocarcinoma of the lung who was found to have a rapidly growing, undiagnosed mass in the left atrium.

## Case presentation

**Fig. 1:**
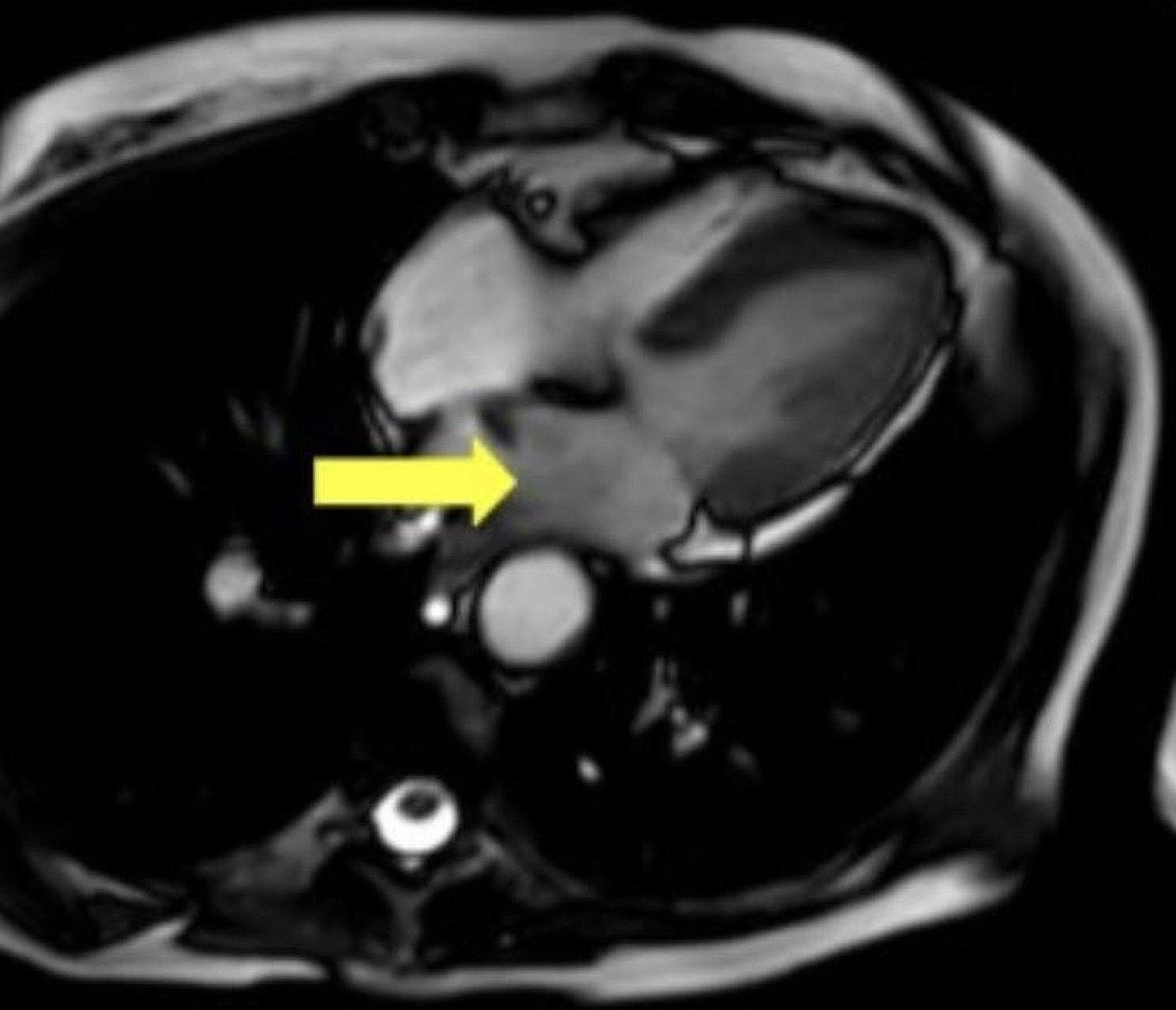
Cardiac magnetic resonance imaging (clip from a balanced steady state free precession [bSSFP] cine sequence) demonstrating a 2 cm X 1.1 cm hypointense lobulated mass lesion arising from the left atrium and interatrial septum at the level of fossa ovalis

**Fig. 2:**
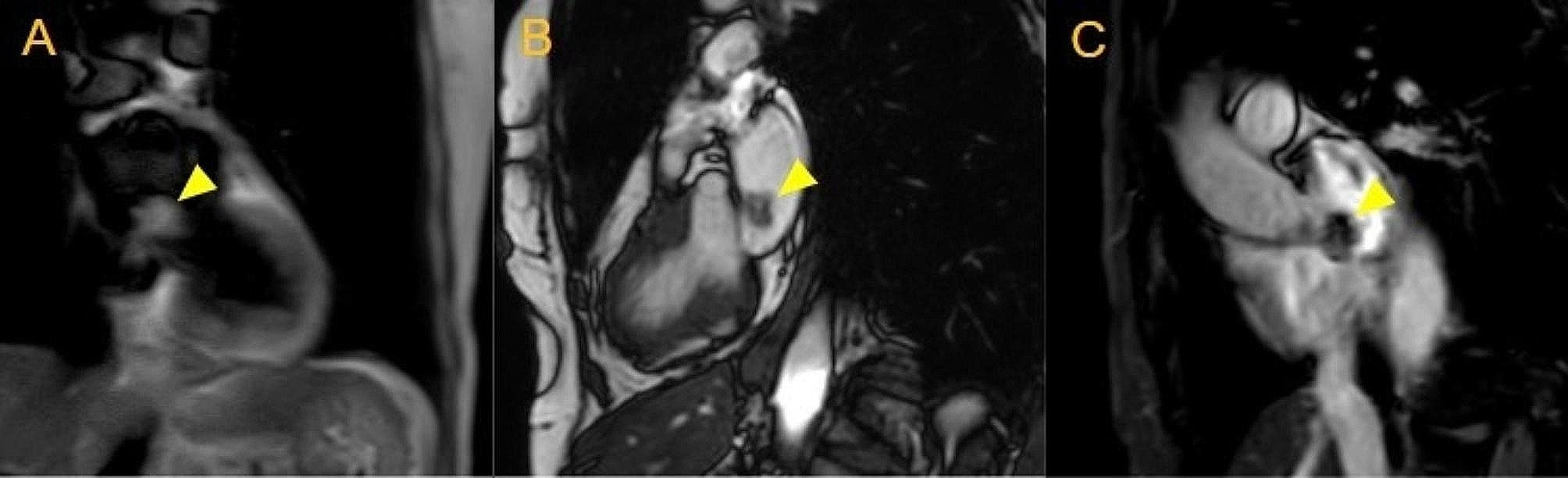
A left atrial mass (yellow arrowhead) is seen appearing isointense to myocardium on a dark blood T1-weighted spin echo coronal view (panel **A**), isointense/mildly hyperintense to myocardium on a balanced steady state free precession 2-chamber view (panel **B**), and heterogeneously hypointense on a late gadolinium-enhanced very high basal short axis view (panel **C**) when assessed by cardiac MRI

A 72-year-old female with a three-year history of recurrent and metastatic adenocarcinoma of the lungs was found to have an undiagnosed mass in the left atrium during chest computed tomography (CT) scan performed to evaluate cancer progression. Further evaluation with cardiac magnetic resonance (MRI) imaging demonstrated a left atrial mass with size of 1.2 cm in width and 1.3 cm in length (Figs. 1 and 2). The location, morphology, and mild enhancement on the perfusion phase as well as on the delayed gadolinium images were suggestive of left atrial myxoma. However, due to the patient’s lung cancer diagnosis and already existing metastasis, concern for further metastasis to the heart was high on the differential. Other modalities such as transthoracic echocardiography (TTE) and transesophageal echocardiography were performed to evaluate the mass.

**Fig. 3:**
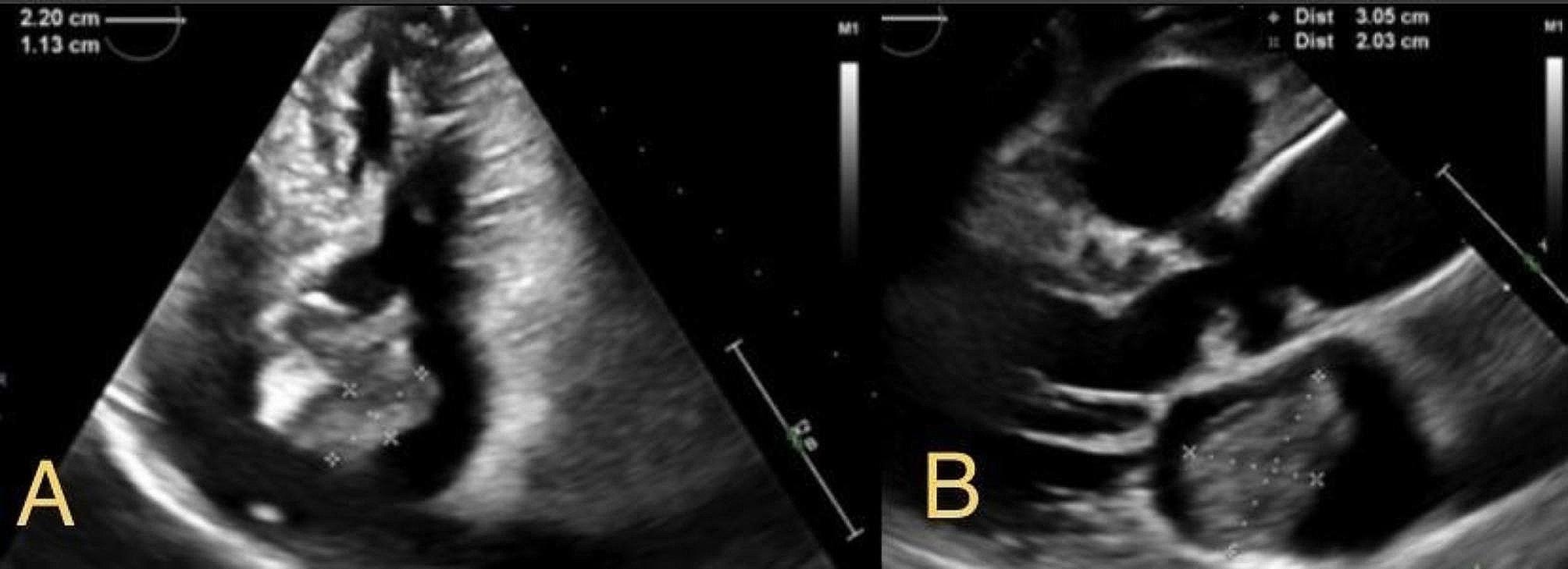
Transthoracic echocardiogram (TTE) images revealing a left atrial cardiac mass. **A** (apical 2-chamber): 2.2 cm X 1.1 cm on initial finding, **B** (parasternal long axis): 3 cm X 2 cm in 14-months from initial discovery

A 3-months follow up with TTE revealed the size of mass now reached 3 cm in width and 1.2 cm in length with a prolapse through the mitral valve (Fig. 3). The patient was referred for surgical evaluation. Unfortunately, the patient was deemed not a surgical candidate as she was very frail. Also, it was not clear if the surgery will be of any benefit as the patient has metastatic disease that was not responding to treatment. Pembrolizumab was added to her initial chemotherapeutic regimen, which included Cisplatin and Pemetrexed. However, the patient was still experiencing cancer progression. The prognosis was unclear and at this time, it was deemed the risk of adverse events during surgery outweighed any benefit. The patient was sent back to the cardio-oncology clinic for surveillance and treatment.

We reasoned that current chemotherapy was likely a major factor in the patient’s frailty. As a result, we decided to allow the patient to finish chemotherapy and immune checkpoint inhibitor therapy, while encouraging the patient to increase physical activity before attempting surgery. The patient was able to increase her physical activity by walking 2 miles a day. We repeated serial TTE during this process to assess progression of left atrial mass.

A 9-month follow-up TTE showed significant growth of the atrial mass with mitral valve obstruction. TTE (Video 1) was repeated in 6-weeks to follow up on the development of the mitral valve prolapse. The mass has grown to 3 cm in width and 2 cm in length. The diastolic flow grade around the mass through the mitral valve was 6 mmHg. Due to this rapid growing nature of the cardiac mass, worsening of diastolic flow obstruction, and the concern for embolism potential, the patient underwent a surgical excision of the mass. This worsening of mitral valve obstruction promoted a multidisciplinary meeting to address the patients mass due to the concern of impending embolism.

The patient was admitted and she underwent comprehensive evaluation in the inpatient setting. She had gained significant strength during the 9-month period and was walking 2–3 miles a day without dyspnea. She had also started on an investigational agent which per the oncology team was promising. The multi-disciplinary team decided that tumor progression and obstruction was more likely to occur than an adverse outcome during surgery. Patient underwent a successful surgery with no complications.

**Fig. 4:**
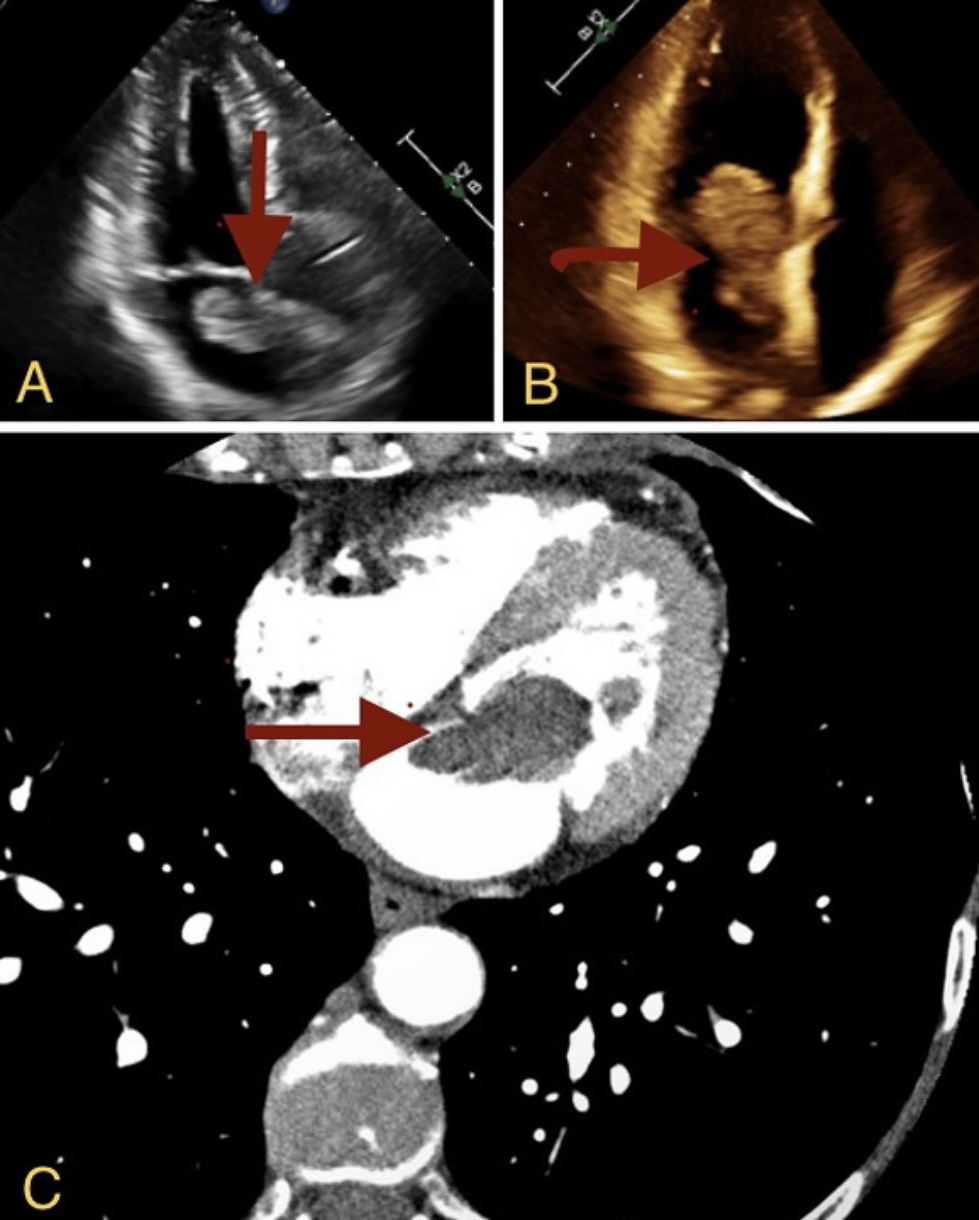
Transthoracic echocardiogram (TTE) displaying the extent of hemodynamic impact from the left atrial mass. (**A**) The mass was initially contained in the left atrium (apical 3-chamber view). (**B**) Follow-up imaging showing prolapse of the mass through the mitral valve into the left ventricle (apical 2-chamber view). (**C**) Computed Tomography (CT) (axial slice through the mitral valve) with contrast also showing the extent of left ventricular obstruction with high risk of rupturing and embolizing with possible catastrophic complications

**Fig. 5:**
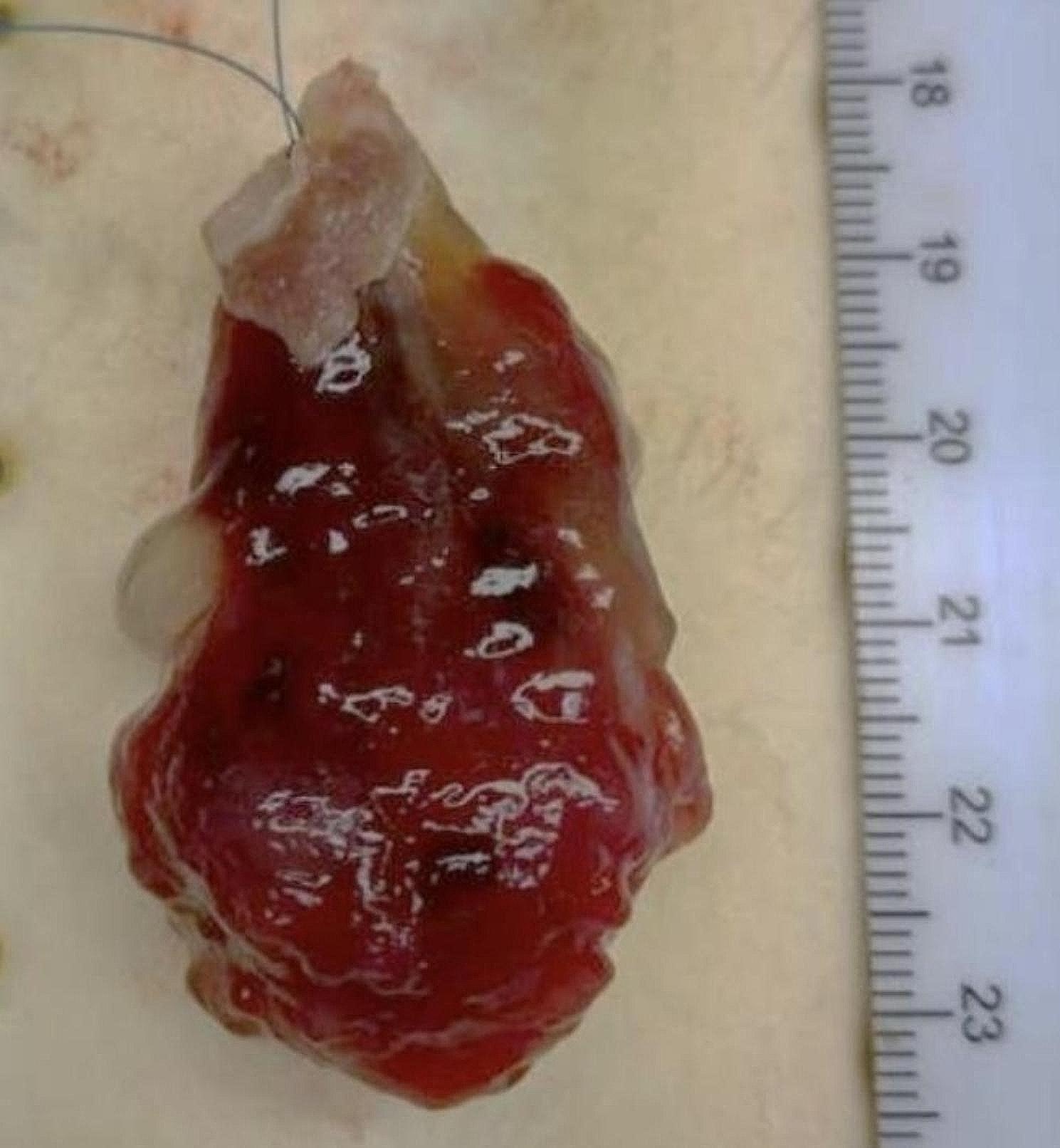
Gross image of a cardiac mass (5 cm in width, 1.5 cm in length, and 3 cm in depth. 1.5 cm in size). Notice the tan-red color and smooth and glistening surface

**Fig. 6:**
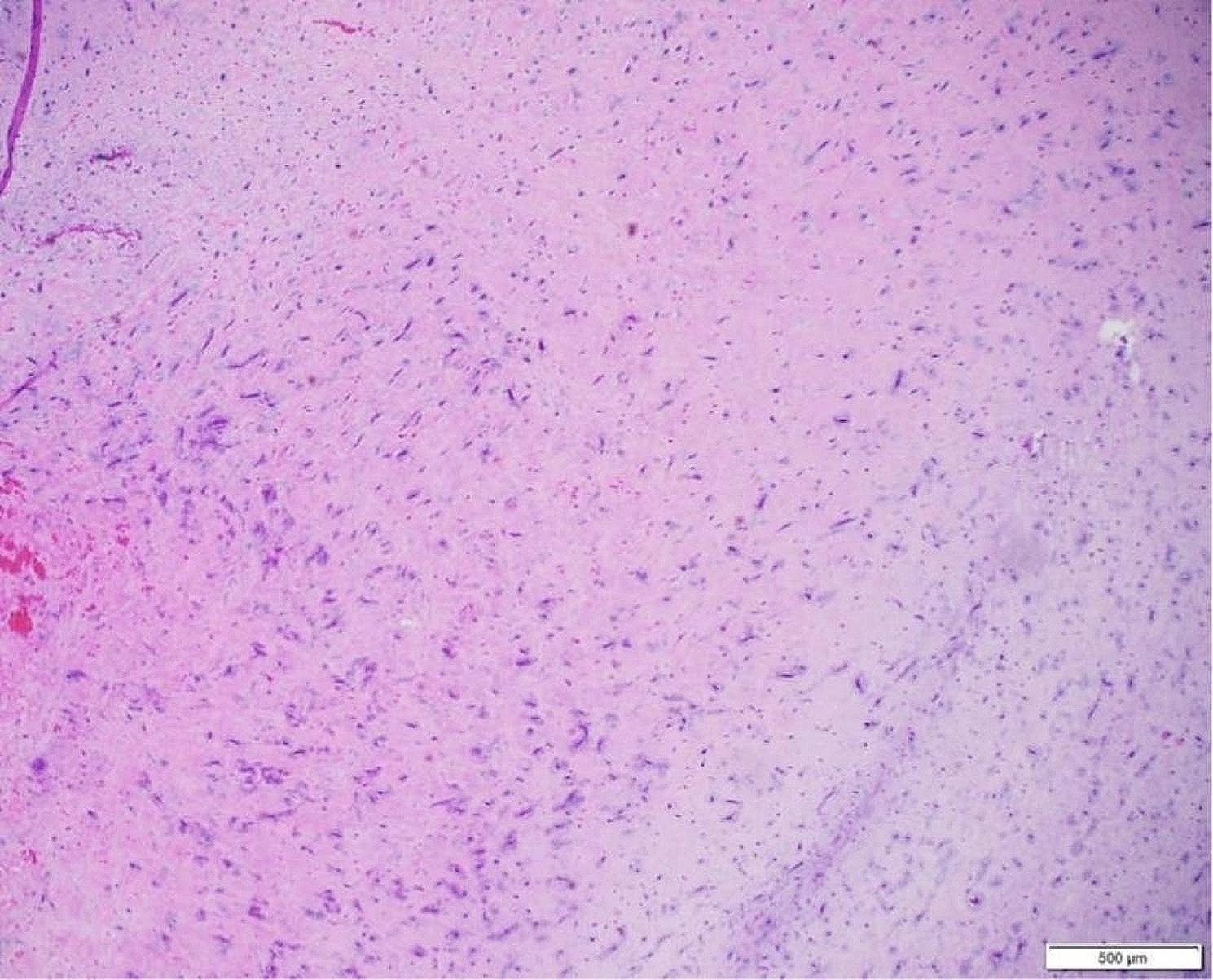
Low power histological examination of a cardiac mass (Hematoxylin and Eosin staining at the magnification of 4X), demonstrating a myxoid background with proliferation of myxoma cells surrounding thin-walled vascular channels

**Fig. 7:**
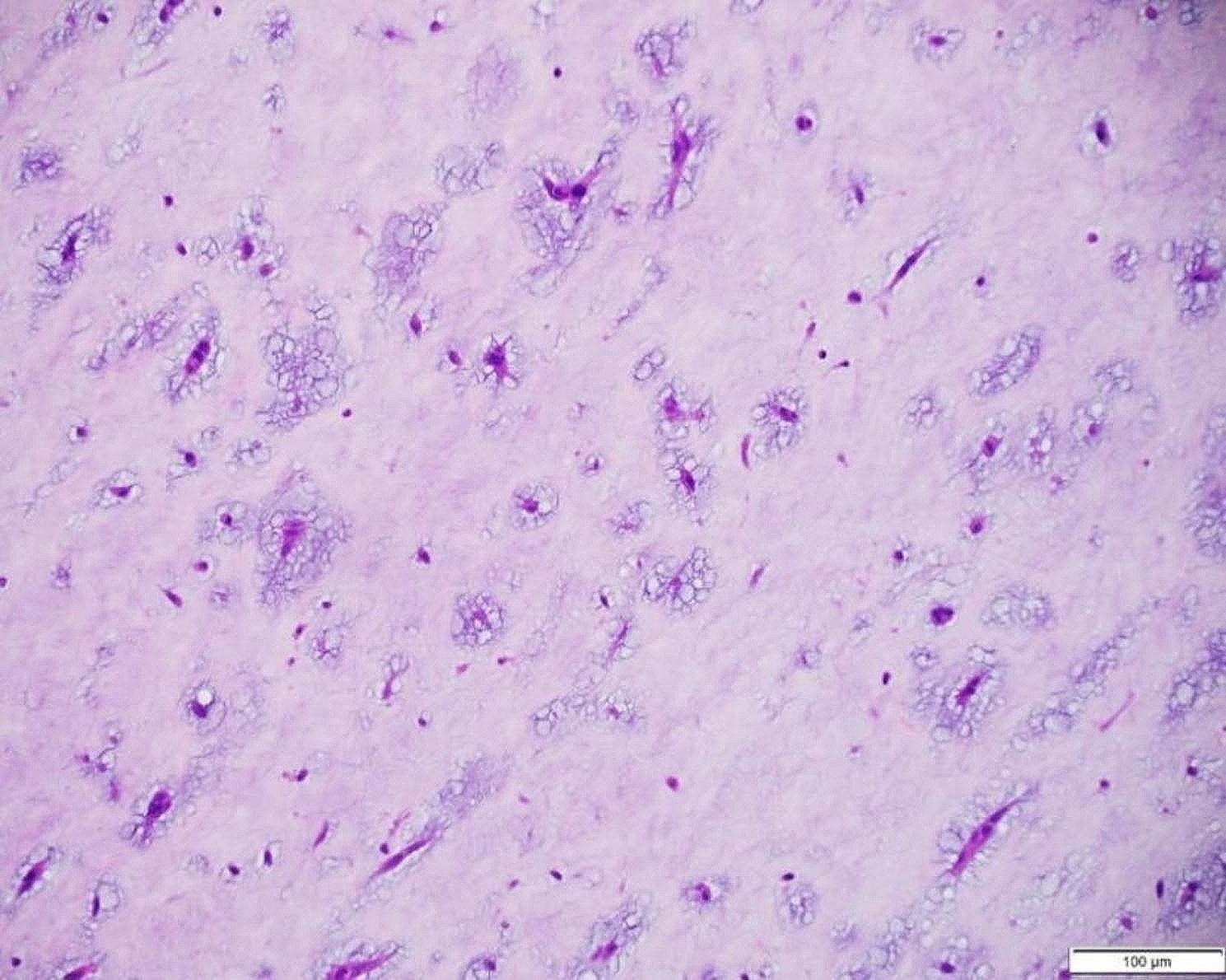
High-power histological examination of a cardiac mass (Hematoxylin and Eosin staining at the magnification of 40X), demonstrating numerous, bland appearing myxoma cells in a perivascular distribution

The cardiac mass was tan-red colored and 5 cm in width, 1.5 cm in length, and 3 cm in depth. 1.5 cm in size (Fig. 4). It had a broad base and several pedicles. Its surface appeared smooth and glistening. Upon examination, its consistency was gelatinous and friable. These gross features were suggestive of cardiac myxoma. Microscopic evaluation revealed the pathognomic characteristics of a myxoma with numerous myxomal cells present in the myxoid stroma (Figs. 5 and 6). A histo-immunological testing showed a positive staining of the tissue sample in both Alcian Blue (AB) and Periodic Acid Schiff (PAS) (Fig. 7).

## Discussion

This case involved a patient with an extensive oncologic history who necessitated complex medical decision making and a multidisciplinary approach in the setting of a newly discovered cardiac neoplasm. Cardiac neoplasms are rare, with an incidence between 0.02 and 2.33% identified by autopsy studies [8, 9]. Most cardiac neoplasms are of metastatic origin. Metastatic secondary cardiac tumors are 20 to 40 times more common than primary cardiac tumors. One study found that the prevalence of primary cardiac tumors was between 0.001 and 0.03% [10]. Fortunately, most primary cardiac tumors (between 75−84.6%) are benign with myxomas being the 2nd most common benign cardiac tumor after papillary fibroelastoma [10, 11]. When a cardiac neoplasm is identified, given the epidemiology of cardiac masses, it is most likely to be of metastatic origin.

On cardiac MRI, both myxomas and metastatic masses tend to display a heterogeneous appearance, with higher signal intensity on T2-weighted sequences compared to T1-weighted sequences. Cine balanced Steady-State Free Precession (bSSFP) sequences can help differentiate myxomas from metastatic masses by characterizing their mobility and attachment, although identifying attachment may be difficult in exceptionally large masses that fill the entire cavity (12–13). Metastatic masses typically involve areas of myocardial-pericardial adhesion [13]. Additionally, after gadolinium injection, a high intensity heterogeneous enhancement is usually seen in metastatic masses. Gadolinium enhancement seen in myxoma is rather moderate in its intensity (12–13). It is challenging to distinguish between primary cardiac neoplasms and metastases based solely on physical exams and imaging studies. Therefore, our recommendation is to refer patients with an unknown cardiac mass, particularly those at a high risk of malignancy, to a cardiology specialty clinic for further evaluation.

In this patient’s case, the clinical concern for cardiac metastasis was especially high given the progressive nature of her pulmonary malignancy combined with the rapidly enlarging nature of the mass. The most common origins of cardiac metastasis include melanoma, carcinoma, which includes lung, breast, esophagus, and rarely colorectal, and hematologic malignancies [1]. In the setting of secondary cardiac masses, primary lung cancer represents 36–39% of cardiac metastasis [14]. Interestingly, despite its anatomic and functional proximity, cardiac metastasis from lung cancer is rarely discovered outside of postmortem pathology (e.g., autopsy studies). This is because secondary cardiac masses from lung cancer infrequently cause clinical signs and symptoms [1]. Similarly, this patient had no cardiac or pulmonary symptoms, and this cardiac mass was discovered incidentally as part of her pulmonary malignancy workup. It was unclear whether it was myxoma, metastasis or another primary tumor. Due to the common practice of surgical removal shortly after diagnosis, there is limited literature available to draw meaningful conclusions about their growth rate [15]. This limited data posed further difficulty in discerning whether the mass is a cardiac myxoma or metastatic mass. We reasoned it was probably not metastatic as they do not usually occur as solitary masses.

Surgical resection remains the most common and effective treatment for both benign and malignant primary cardiac tumors, given its safety and high success rate [16]. For patients who may not be suitable candidates for surgery, Magnetic Resonance Guided Radiotherapy (MRgRT) could be considered as an alternative treatment option. MRgRT offers direct target visualization during treatment, allowing higher-dose administration with reduced harm to adjacent healthy tissue [17].

The progression of the mass created a clinical urgency and the need to reevaluate embolic risk versus the perioperative risk in the patient. Rupture and embolism of cardiac myxoma are significant complications, occurring in 30–50% of patients [18]. The rate of such complications can vary greatly for cardiac metastasis, depending on the type of primary tumor. To prevent such an outcome, the patient was admitted to the cardiac intensive care unit where she underwent a multidisciplinary evaluation that included the perspectives of oncology, cardiology, cardiothoracic surgery, and interventional cardiology.

Using this collaborative approach, the decision was made to pursue surgical mass excision, which revealed a left atrial myxoma rather than pulmonary metastasis. In the absence of a multidisciplinary approach, it can easily be deduced that this patient would have had a poor outcome, likely in the form of an embolic event, as she had multiple clinical and epidemiological reasons to assume her mass was metastatic in origin and inoperable. Given the rarity of primary cardiac tumors, a collaborative approach was vital in making an accurate diagnosis, and deciding on the appropriate timing to attempt surgery to obtain satisfactory outcome in the realization that data we had was incomplete and with the need to make the best decision possible.

## Conclusion

Differentiating between distinct types of masses can pose a challenge to the treatment team especially in the setting of malignancy. While myxomas are the most common left atrial masses seen, due to the rapid enhancement and eventual obstruction in the setting of recurrent malignancy this case warranted tissue diagnosis. This case demonstrates the importance of multidisciplinary approach and discussions to provide appropriate treatment in a complex clinical scenario.

### Electronic supplementary material

Below is the link to the electronic supplementary material.

**Supplementary Material 1: Video 1.** Transthoracic echocardiogram (TTE) revealing a cardiac mass (3 cm in width and 2 cm in length) with a prolapse through the mitral valve. The diastolic flow grade around the mass through the mitral valve was 6 mmHg. Left ventricular ejection flow was measured to be 58%

## Data Availability

Data and materials are stored in the Department Drive can be accessed upon a request.
